# Viral Respiratory Infections in Djibouti: Insights from Two Years of Pilot Surveillance

**DOI:** 10.3390/v17121525

**Published:** 2025-11-21

**Authors:** Hamda Omar Assoweh, Mohamed Houmed Aboubaker, Mamadou Malado Jallow, Sahal Darar Dirir, Issa Gnasse, Filsan Daher Aouled, Daouda Diallo, Amina Ahmed Ibrahim, Ndaraw Diack, Mouchtak Nabil Ahmed, Ndongo Dia, Makhtar Camara

**Affiliations:** 1 Laboratoire de Diagnostic, Caisse Nationale de Sécurité Sociale (CNSS), Djibouti 98230, Djibouti; assowehomarhamda@gmail.com (H.O.A.); houmedm@yahoo.fr (M.H.A.); sahal.darar@cnss.dj (S.D.D.); fdaheraouled@gmail.com (F.D.A.); 77763381a@gmail.com (A.A.I.); mouchetek.nabil@cnss.dj (M.N.A.); 2 Laboratoire de Biologie Médicale Mer Rouge, Djibouti 41170, Djibouti; 3 Département de Virologie, Institut Pasteur de Dakar, Dakar BP 5005, Senegal; mamadoumalado.jallow@pasteur.sn (M.M.J.); issalayegnasse@gmail.com (I.G.); daouda1990.diallo@gmail.com (D.D.); ndaraw.diack@pasteur.sn (N.D.); 4 Département Pharmacie, Faculté de Médecine, Université Cheikh Anta DIOP de Dakar, Dakar BP 5005, Senegal; makhtar.camara@ucad.edu.sn

**Keywords:** viral respiratory infections, surveillance, epidemiology, influenza-like illness, acute respiratory infections, Djibouti

## Abstract

Viral respiratory infections (VRIs) remain a leading cause of global morbidity and mortality. In Djibouti, diagnostic capacity for respiratory viruses is extremely limited, and little is known about the epidemiology of VRIs in the country. To address this gap, we conducted a prospective study aimed at monitoring and describing the epidemiological and virological features of VRIs over a two-year period in Djibouti. The study was carried out between January 2023 and December 2024 in seven healthcare centers. Nasopharyngeal and/or oropharyngeal swabs were collected from consenting patients presenting with influenza-like illness (ILI) or acute respiratory infections (ARI). VRIs were diagnosed using a multiplex real-time reverse transcription polymerase chain reaction (rRT-PCR) assay targeting 16 respiratory viruses. Among the 647 samples tested, at least one respiratory virus was detected in 133 (20.6%). Influenza (6.6%), rhinovirus (5.9%), and SARS-CoV-2 (3.4%) were the most frequently detected pathogens. Adult aged 26–50 years (40.8%) were the most affected, followed by those aged 15–25 years (30.4%). None of the respiratory viruses displayed a clear seasonal pattern. These findings demonstrate the co-circulation of multiple respiratory viruses among ILI/ARI patients in Djibouti and provide the first baseline data to inform national surveillance strategies. The results underscore the need to strengthen routine laboratory-based surveillance, enhance diagnostic capacity, and integrate molecular monitoring into national public health policies for better preparedness against emerging respiratory threats.

## 1. Introduction

Viral respiratory infections (VRIs) are among the most common infectious diseases and remain a leading cause of global morbidity and mortality [1]. They can cause recurrent seasonal epidemics and, occasionally, pandemics such as the severe acute respiratory syndrome (SARS) in 2003, the H1N1 influenza pandemic in 2009, the Middle East respiratory syndrome (MERS) in 2012, and the severe acute respiratory syndrome coronavirus 2 (SARS-CoV-2) pandemic in 2019. These events have resulted in considerable health and economic impacts worldwide. The public health risks and potential for large-scale transmission associated with VRIs are now greater than ever. Contributing factors include population growth and urbanization, the rapid expansion of global travel, civil conflicts and migration, climate change, and other forms of human-induced environmental degradation, all of which have increased the likelihood of future pandemics [2].

VRIs exhibit similar clinical manifestations, ranging from the common cold to severe acute respiratory distress syndrome (ARDS) [3,4]. The causative agents belong to several viral families and primarily target the respiratory system; they can infect both the upper and lower respiratory tract and often display a seasonal pattern profile. They are highly contagious and transmitted either through direct contact with respiratory secretions or indirectly via contaminated objects or surfaces [3]. Children under five years of age are disproportionately affected, and the burden is particularly high in low- and middle-income countries (LMICs) [4,5]. Approximately 97% of severe VRIs are reported in low-income countries, with nearly 70% of cases occurring in South Asia and sub-Saharan Africa [3,5]. In these regions, the etiology of respiratory infections is often poorly understood due to the diversity of respiratory pathogens, limited diagnostic resources, the overlapping clinical features of VRIs, and confusion with other febrile tropical illnesses. These challenges make the management of such infections particularly difficult, especially in children [4,6].

In Djibouti, a small country in the horn of Africa, data on VRIs are scarce, with most investigations limited to the COVID-19 pandemic [7,8]. To fill this gap, we conducted a two-year prospective study to monitor and describe the epidemiological and virological characteristics of viral respiratory infections in Djibouti.

## 2. Materials and Methods

### 2.1. Study Location and Setting

The Republic of Djibouti is a country located in the Horn of Africa with an area of 23,200 km^2^. It is bordered by Somalia to the south, Ethiopia to the southwest, Eritrea to the north, and the Red Sea and the Gulf of Aden to the east. Djibouti is a multi-ethnic nation with a population of 1,066,809 according to the national census conducted on 20 May 2024 (Institut National de la Statistique de Djibouti, INSTAD, 2024, https://instad.dj/, accessed on 26 September 2025). The capital city is also named Djibouti.

This prospective study was conducted between January 2023 and December 2024 in a country with no prior history of respiratory infection surveillance. The surveillance focused on monitoring respiratory syndromes, namely influenza-like illness (ILI) and acute respiratory infections (ARIs). Patient recruitment was carried out in seven healthcare facilities (hospitals and health centers), all located in Djibouti city: Peltier General Hospital, Cheikho Hospital, CNSS Health Center 2, PK12 Polyclinic, Khor Bourhan Polyclinic, Farah-Had Polyclinic, and Einguella Polyclinic. Authorization to conduct respiratory infection surveillance at these sites was obtained from the Ministry of Health of the Republic of Djibouti.

At each healthcare center, trained physicians identified patients with ILI or ARI, following the WHO case definitions. ILI was defined as an acute respiratory infection with: measured fever of ≥38 °C, and cough with onset within the last 10 days. ARI was defined as an acute respiratory infection with onset within the past 10 days, including at least one of the following symptoms: cough, sore throat, shortness of breath, or coryza, with or without fever [9]. Informed written consent was obtained from patients or their guardians before enrollment. Clinicians subsequently collected patient information, including demographic data (age, sex) and clinical symptoms such as fever, cough, wheezing, rhinorrhea, headache, dyspnea, myalgia, arthralgia, and diarrhea.

### 2.2. Collection and Transportation of Clinical Samples

Nasopharyngeal and/or oropharyngeal swabs were collected from consenting patients of all ages attending healthcare centers participating in this pilot surveillance and meeting the case definition. The swabs were placed in universal viral transport medium (VTM) (Becton Dickinson and company, Franklin Lakes, NJ, USA) and immediately stored at −80 °C at the National Institute of Public Health of Djibouti (INSPD) until transport. Samples, along with the corresponding case-based forms, were then shipped to the Senegalese National Influenza Center (NIC) at the Institut Pasteur de Dakar (IPD) for the screening of respiratory viruses.

For transportation, frozen samples were placed in hermetically sealed secondary packaging with ice packs inside an IATA-compliant triple packaging system and sent via commercial flight to Dakar (within approximately 10 h). Upon arrival at IPD, samples were immediately stored at −80 °C until analysis.

### 2.3. Nucleic Acid Extraction and Screening of Respiratory Viruses

Total nucleic acids (DNA/RNA) were extracted from 200 μL of swab suspension in VTM using the QIAamp Viral RNA kit (QIAGEN, Valencia, CA, USA) according to the manufacturer’s instructions. Nucleic acids were eluted in 60 µL of elution buffer and immediately used for respiratory virus screening. Extracted RNA and DNA from all samples were tested for 16 respiratory viruses, including Influenza A virus, Influenza B virus, Respiratory syncytial virus (RSV), SARS-CoV-2, adenovirus, Human metapneumovirus (HMPV), Human coronaviruses (229E, NL63, OC43), Human parainfluenza viruses (PIV1, -2, -3 and -4), rhinoviruses (RVs), enteroviruses (EVs) and Human bocaviruses (HBoVs), using a multiplex real-time reverse transcription polymerase chain reaction (rRT-PCR) assay with the Allplex™ Respiratory Full Panel Assay (Seegene, Seoul, Republic of Korea), as previously described [10]. Real-time RT-PCR was performed on a CFX96™ platform (Bio-Rad, Hercules, CA, USA) and analyzed with Seegene’s Viewer software(Ver 3.24.000). The Allplex™ Respiratory Full Panel Assay software automatically generates amplification curves up to a Ct value (cycle threshold values) of 42; however, for the interpretation of results, only samples with Ct values ≤ 37 for each target virus were considered positive.

### 2.4. Statistical Analysis

Statistical analyses were performed using R software (R.3.0.1 version). Fisher’s exact test or the chi-square (χ^2^) test was used to compare demographic and clinical characteristics between the study population and positive cases. A *p*-value < 0.05 was considered statistically significant.

## 3. Results

### 3.1. Clinical and Demographic Characteristics of Enrolled Patients

During the 2-year pilot surveillance, nasopharyngeal swabs were collected from 647 patients with respiratory syndromes, aged 1 month to 89 years (mean age: 25.5 years; median age: 24 years). Most of these patients were enrolled at Farah-had polyclinic (47.4%, 307/647) and CNSS Health Center 2 (42.3%, 273/647). The remaining healthcare centers contributed fewer specimens, ranging from 14 samples at Peltier General Hospital to 4 samples at Khor Bourhan Polyclinic (0.6%). The majority of patients were recruited in 2024 (68.6%, 444/647), while 31.4% (203/647) were enrolled in 2023. Overall, 57.8% (374/647) of the patients were male and 42.2% (273/647) female, giving a sex ratio of 1.36. Adults aged 26–50 years were the most represented group (34.9%, 226/647), followed by those aged 15–25 years (22.9%, 148/647), children under 5 years (17.2%, 111/647) and patients aged 6–14 years (14.7%, 95/647). Elderly patients (>50 years) represented 10.4% (67/647). At consultation, the most frequent clinical diagnoses were acute bronchitis (15.1%), acute bronchiolitis (14.7%) and pneumonia (14.5%). Other diagnoses included asthma exacerbation (3.6%), acute otitis (2.3%), rhinitis (2.5%), pharyngitis (2.9%) and acute sinusitis (2.5%). Regarding symptoms, the majority presented with cough (97.5%), fever (86.4%), headache (49.9%) and myalgia (45.4%). Dyspnea, a symbol of severe respiratory infection, was observed in 7.1% of patients (Table 1).

### 3.2. Detection of Respiratory Viruses

**Figure 1:**
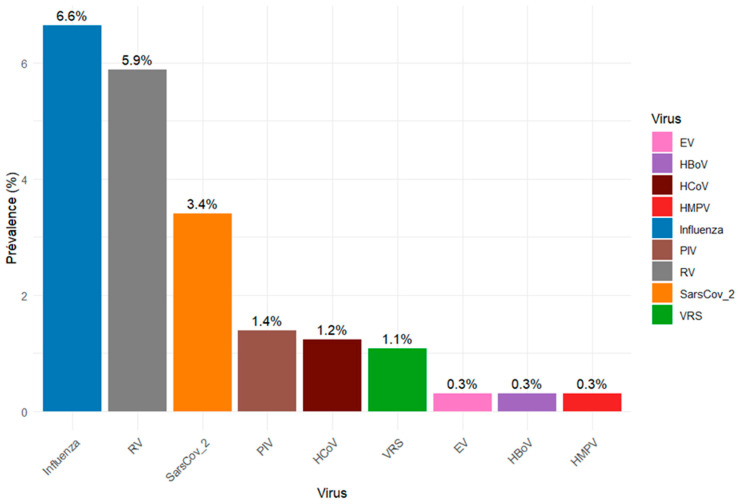
Proportion of respiratory viruses detected during the study period in Djibouti.

Of the 647 specimens tested during the study period, at least one respiratory virus was detected in 133 (20.6%). Influenza viruses were the most frequently identified, with an overall detection rate of 6.6% (43/647), followed by rhinoviruses (5.9%, 38/647) and SARS-CoV-2 (3.4%, 22/647). Of the 43 influenza viruses detected, type B accounted for 58.1% (25/43), whereas type A represented 41.9% (18/43). Other viruses were detected at lower frequencies, including parainfluenza viruses (1.4%, 9/647), human coronaviruses (1.2%, 8/647), respiratory syncytial virus (1.1%, 7/647), enteroviruses (0.3%, 2/647), bocavirus (0.3%, 2/647) and human metapneumovirus (0.3%, 2/647) (Figure 1).

Single viral infections were observed in 93.2% (124/133) of cases, while co-infections with multiple viruses were identified in 6.8% (9/133). These included two cases of SARS-CoV-2/HCoV-OC43 coinfection, one case of influenza A/SARS-CoV-2 coinfection, one case of influenza A/rhinovirus coinfection, one case of influenza B/SARS-CoV-2 coinfection, one case of influenza B/rhinovirus coinfection, one case of influenza B/HCoV-OC43 coinfection, one case of HMPV/PIV3 coinfection, and one case of triple infection involving influenza B, SARS-CoV-2, and HCoV-OC43.

### 3.3. Clinical and Demographic Characteristics of Patients with Confirmed VRI

Demographic characteristics and clinical parameters of patients with respiratory viral infections are summarized in Table 2. During the study period, patients with confirmed viral infections ranged in age from 8 months to 70 years, with a mean age of 23.6 years and a median age of 22 years. Although statistical analyses did not reveal significant differences due to limited sample size, influenza (53.5%), rhinovirus (60.5%), and RSV (71.4%) infections were more frequent in male patients, whereas SARS-CoV-2 (54.5%), PIV (55.6%), HCoV (62.5%) and enterovirus (100%) infections were more common in females.

With respect to age distribution, viral infections were detected most frequently in adult patients aged 26–50 years (42.8%), followed by those aged 15–25 years (30.1%). However, rhinovirus showed the highest detection rate in patients aged 15–25 years (42.1%). Interestingly, for RSV typically associated with pediatric infections, almost all cases in this study were found in adults aged 26–50 years (85.7%).

Considering the association between clinical diagnoses and detected viruses, influenza (25.6%), SARS-CoV-2 (22.7%), RSV (42.8%), and HCoV (25%) infections were more often detected in patients with pneumonia, whereas rhinovirus (18.4%) and PIV (44.4%) infections were predominantly observed in patients with acute bronchiolitis. Regarding clinical symptoms, all detected viruses presented with similar features, most commonly fever, cough, headache and myalgia.

### 3.4. Temporal Distribution of Viral Confirmed Cases

**Figure 2:**
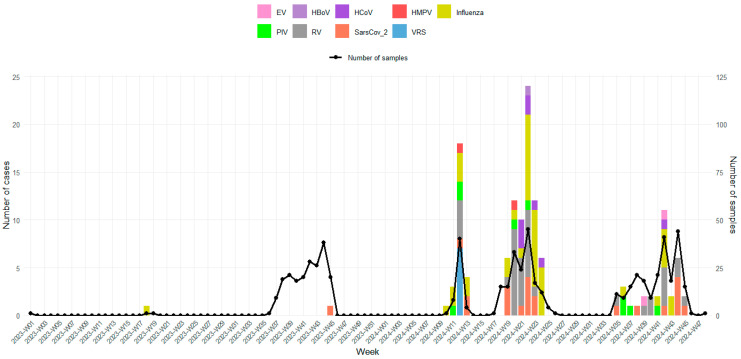
Weekly distribution of detected respiratory viruses during the study period (2023–2024). Bars represent the number of positive cases for each week, and the curve represents the number of samples tested.

The temporal distribution of detected viruses on a weekly basis was also investigated and is shown in Figure 2. During the study period, nearly all confirmed respiratory virus cases were detected in 2024 (98.5%, 131/133). In 2023, only two viruses were identified: one influenza case in week 18 (May) and one SARS-CoV-2 case in week 45 (November). This extremely low viral detection rate in 2023 can be explained by delays in sample collection, as 98.5% (200/203) of the samples were collected between September and November 2023. None of the respiratory viruses displayed a clear seasonal pattern. Indeed, multiple respiratory viruses co-circulated, with distinct peaks of activity. Influenza showed several pronounced peaks, the most significant occurring between weeks 21–24 (May–June), coinciding with an increased number of specimens tested. Smaller influenza peaks were also observed in early 2024 (weeks 10–13) and late 2024 (weeks 41–43). Rhinovirus was consistently detected across multiple weeks, with moderate peaks overlapping influenza activity; the highest detection rates occurred during weeks 20–22 (May). SARS-CoV-2 infections were identified intermittently without major peaks, while all RSV cases were detected in week 12. Other respiratory viruses, including Human coronaviruses (HCoVs), parainfluenza viruses (PIVs), human metapneumovirus (HMPV), bocavirus (HBoV) and enterovirus (EV), were detected sporadically at lower levels.

## 4. Discussion

In Djibouti, diagnostic capacity for respiratory viruses remains extremely limited, and little is known about the epidemiology of viral respiratory tract infections (RTIs) in the country despite their well-documented clinical and economic impact [11,12,13]. This two-year investigation of viral respiratory infections (VRIs) among patients with ILI/ARI, conducted in collaboration with seven healthcare centers, represents the most comprehensive study of its kind in Djibouti. Our findings showed that 20.6% of the 647 samples tested during the study period were positive for at least one of the 16 targeted respiratory viruses, although the distribution of pathogens varied. The overall viral detection rate reported here is considerably lower than those from previous etiological studies conducted in similar settings, including Senegal (77.8%), Alaska (90%), Suriname (92%), Thailand (58.6%), Kenya (49.8%), Gabon (61.4%), and The Gambia (72.1%) [14,15,16,17,18,19,20]. Nevertheless, our detection rate is comparable to that reported in a study conducted in Cameroon between January 2017 and January 2018, where VRIs accounted for 22.1% of LRTIs in hospitalized adults [21]. Consistent with previous reports from Niang et al. in Senegal [22], Chadha et al. in India [23] and Bellei et al. in Brazil [24], influenza viruses and rhinoviruses were the most frequently detected agents. However, unlike the seasonal influenza patterns described in many tropical countries [25,26,27], influenza activity in our study peaked between May and June. Rhinoviruses were also commonly detected, with peaks overlapping those of influenza. This is consistent with the findings of Boivin et al. [28], who reported rhinoviruses as a frequent cause of significant respiratory and systemic illness in adults during influenza season across diverse regions, including Canada, Hong Kong, and several European countries. In contrast, a study by Lekana-Douki et al. in Gabon found AdV (17.5%), PIVs (16.8%), EV (14.7%) and RSV (13.5%) to be the most prevalent viral pathogens among ILI patients [18]. In our study, these viruses were detected at much lower levels, ranging from 0% for AdV to 1.4% for PIVs. Similarly, while Vega-Piris et al. [29] recently reported SARS-CoV-2 as the leading viral pathogen (62%) among hospitalized adults ≥ 65 years in Spain (October 2021–May 2024), our study detected SARS-CoV-2 only intermittently and at a much lower rate (3.4%). Mixed viral infections were also relatively uncommon (6.8%), a rate comparable to that of Pierangeli et al. in Italy [30], but much lower than reported in several other studies [31,32,33]. Overall, the comparatively low viral detection rate in our study may be explained by several factors, including differences in case selection criteria, patient age distribution, seasonal timing of sample collection, regional variations in virus circulation and healthcare-seeking behavior.

With regard to age distribution, VRIs were identified across all age groups, as reported elsewhere [23,24]. However, most cases (74.4%; 99/133) occurred in adults (≥18 years), with over half of them aged 26–50 years old (51.5%; 51/99). This contrasts with the large body of evidence indicating that children, particularly those under five, are usually the most affected group [32,34,35]. In our study, children under five represented only 10.4% of VRI cases, likely reflecting a bias in the composition of the study population, where adults (≥18 years) constituted 63.5%. Rhinovirus detection was highest in patients aged 15–25 years (42.1%), consistent with previous reports identifying it as a major cause of ARI in adolescents and young adults [28,36]. Interestingly, RSV was almost exclusively detected in adults aged 26–50 years, in contrast to its well-established role as a leading pathogen in infants and young children [10,37,38,39,40]. The discrepancy of the distribution of respiratory viruses across age groups compared with other studies, particularly for RSV, is most likely explained by a bias in the composition of our study population. Indeed, infants aged ≤ 1 year, who are traditionally the most affected group by RSV infections [9], represented only 6.8% (44/647) of our enrolled participants. As a result, the age structure of the recruited population may have underestimated the true RSV burden, since the surveillance sites included in our study primarily serve older children and adults. Other contributing factors could include differences in healthcare-seeking behavior, the seasonal timing of sample collection, and the fact that sentinel sites were not originally designed to capture the full pediatric burden of RSV. This finding underscores the need to strengthen and expand surveillance activities, particularly among pediatric populations and to integrate genomic characterization to better understand circulation and evolution of respiratory viruses in Djibouti.

Clinical analysis showed that most patients with VRI in this study presented with lower respiratory tract infections (LRTIs). These observations are consistent with previous reports linking RSV, influenza and coronaviruses to lower respiratory tract disease, and identifying rhinovirus and PIV as frequent causes of bronchiolitis [10,30,41]. Regarding clinical presentation, the majority of viral infections were characterized by non-specific symptoms such as fever, cough, headache, and myalgia, consistent with the overlapping spectrum of respiratory viruses, which complicates etiological diagnosis based on clinical features alone.

This study has several limitations. First, the relatively small number of samples limits the robustness of our findings. Expanding the number of specimens and increasing the participation of healthcare sites would provide a more comprehensive picture of VRIs in Djibouti. Second, the limited recruitment period did not allow us to fully assess the seasonality of viral infections, an important component of respiratory virus epidemiology. Finally, the absence of systematic testing for bacterial pathogens restricted our ability to evaluate bacterial-viral coinfections and their clinical impact.

Despite these limitations, this study provides valuable insights into the epidemiology and clinical presentation of VRIs in Djibouti, a setting where such data remain scarce, and underscore the need to strengthen national surveillance and capacity-building efforts. Expanding sentinel surveillance to include pediatric sites, enhancing laboratory diagnostic and genomic capacities, and reinforcing training for healthcare workers in specimen collection and data management would improve early detection and response to respiratory virus outbreaks. Such initiatives would also facilitate Djibouti’s integration into regional and global surveillance networks, thereby strengthening epidemic preparedness.

## 5. Conclusions

In summary, this study provides the first comprehensive description of the epidemiological and virological features of viral respiratory infections in Djibouti. Almost all detection of VRI cases occurred in 2024 (98.5%), whereas only two cases (1.5%) were detected in 2023. Influenza, rhinovirus and SARS-CoV-2 were the most frequently detected viruses, predominantly in adults, with no clear seasonal trend observed. These findings highlight critical gaps in national surveillance and underscore the need to establish and strengthen ILI and SARI monitoring system, expand testing capacity, and enhance laboratory and genomic diagnostics. Strengthening these capacities will improve early detection, guide vaccination and prevention strategies, and support evidence-based public health decision-making in Djibouti.

## Figures and Tables

**Table 1:** Clinical and Demographic Characteristics of patients enrolled with ARI/ILI in Djibouti, 2023–2024.

Health Centers	Peltier General Hospital	Cheikho Hospital	CNSS Health Center 2	PK12 Polyclinic	Khor Bourhan Polyclinic	Farah-Had Polyclinic	Einguella Polyclinic	Total
Samples Collected	N = 34	N = 14	N = 273	N = 5	N = 4	N = 307	N = 10	N = 647
Gender, n (%)								
Female	13 (38.2)	7 (50)	108 (39.6)	3 (60.0)	2 (50)	136 (44.3)	4 (40.0)	273 (42.2)
Male	21 (61.8)	7 (50)	165 (60.4)	2 (40.0)	2 (50)	171 (55.7)	6 (60.0)	374 (57.8)
Age group, n (%)								
[0–5]	0 (0.0)	0 (0.0)	104 (38.1)	0 (0.0)	1 (25.0)	6 (2.0)	0 (0.0)	111 (17.2)
[6–14]	0 (0.0)	0 (0.0)	54 (19.8)	1 (20.0)	0 (0.0)	40 (13.0)	0 (0.0)	95 (14.7)
[15–25]	11 (32.4)	7 (50)	25 (9.1)	2 (40.0)	3 (75.0)	97 (31.6)	3 (30.0)	148 (22.9)
[26–50]	17 (50.0)	7 (50)	66 (24.2)	1 (20.0)	0 (0.0)	129 (42.0)	6 (60.0)	226 (34.9)
>50	6 (17.6)	0 (0.0)	24 (8.8)	1 (20.0)	0 (0.0)	35 (11.4)	1 (10.0)	67 (10.3)
Diagnostic, n (%)								
URTI	12 (35.3)	0 (0.0)	33 (12.1)	0 (0.0)	1 (25)	21 (6.8)	0 (0.0)	67 (10.4)
LRTI	15 (44.1)	0 (0.0)	140 (51.3)	0 (0.0)	3 (75)	129 (42)	0 (0.0)	287 (44.3)
Others	7 (20.6)	14 (100)	100 (36.6)	5 (100)	0 (0.0)	157 (51.1)	10 (0.0)	293 (45.3)
Symptoms, n (%)								
Fever	23 (67.6)	14 (100)	228 (83.5)	5 (100)	3 (75.0)	276 (89.9)	10 (100)	559 (86.4)
Cough	32 (94.1)	14 (100)	267 (97.8)	5 (100)	3 (75.0)	300 (97.7)	10 (100)	631 (97.5)
Headache	18 (52.9)	14 (100)	96 (35.2)	5 (100)	2 (50)	178 (58.0)	10 (100)	323 (49.9)
Myalgia	15 (44.1)	14 (100)	82 (30.0)	5 (100)	2 (50)	166 (54.1)	10 (100)	294 (45.4)
Dyspnea	3 (8.8)	4 (28.6)	12 (4.4)	2 (40.0)	1 (25.0)	23 (7.5)	1 (10.0)	46 (7.1)

URTI = Upper respiratory tract infection; LRTI = Lower respiratory tract infection.

**Table 2:** Demographic and clinical characteristics of patients with confirmed VRI in Djibouti, 2023–2024.

Viruses	Total Tested	VRI Cases	Influenza	Rhinovirus	SARS-CoV-2	PIV	HCoV	RSV	Bocavirus	Enterovirus	HMPV
Number Positives	N = 647	[N = 133]	[N = 43]	[N = 38]	[N = 22]	[N = 9]	[N = 8]	[N = 7]	[N = 2]	[N = 2]	[N = 2]
Gender, n (%)											
Female	273 (42.2)	63 (47.4)	20 (46.5)	15 (39.5)	12 (54.5)	5 (55.6)	5 (62.5)	2 (28.6)	1 (50.0)	2 (100)	1 (50.0)
Male	374 (57.8)	70 (52.6)	23 (53.5)	23 (60.5)	10 (45.5)	4 (44.4)	3 (37.5)	5 (71.4)	1 (50.0)	0 (0.0)	1 (50.0)
Age group, n (%)											
[0–5]	111 (17.1)	13 (9.8)	1 (2.3)	4 (10.5)	3 (13.6)	1 (11.1)	1 (12.5)	0 (0.0)	1 (50.0)	2 (100)	0 (0.0)
[6–14]	95 (14.7)	8 (6.0)	4 (9.3)	2 (5.3)	1 (4.5)	0 (0.0)	1 (12.5)	0 (0.0)	0 (0.0)	0 (0.0)	0 (0.0)
[15–25]	148 (22.9)	40 (30.1)	13 (30.2)	16 (42.1)	5 (22.7)	2 (22.2)	3 (37.5)	1 (14.3)	0 (0.0)	0 (0.0)	0 (0.0)
[26–50]	226 (34.9)	57 (42.8)	21 (48.8)	13 (34.2)	8 (36.4)	4 (44.4)	3 (37.5)	6 (85.7)	0 (0.0)	0 (0.0)	1 (50.0)
>50	67 (10.4)	15 (11.3)	4 (9.3)	3 (7.9)	5 (22.7)	2 (22.2)	0 (0.0)	0 (0.0)	1 (50.0)	0 (0.0)	1 (50.0)
Clinical diagnosis, n (%)											
URTI	67 (10.4)	10 (7.5)	2 (4.6)	3 (7.9)	3 (13.6)	1 (11.1)	0 (0.0)	0 (0.0)	0 (0.0)	0 (0.0)	1 (50.0)
LRTI	287 (44.3)	67 (50.4)	23 (53.5)	20 (52.6)	10 (45.4)	6 (66.7)	4 (50.0)	5 (71.4)	0 (0.0)	2 (100)	1 (50.0)
Others	293 (45.3)	56 (42.1)	18 (41.9)	15 (39.5)	9 (41.0)	2 (22.2)	4 (50.0)	2 (28.6)	2 (100)	0 (0.0)	0 (0.0)
Symptoms, n (%)											
Fever	559 (86.4)	123 (92.5)	39 (90.7)	36 (94.7)	19 (86.4)	9 (100)	7 (87.5)	7 (100)	2 (100)	2 (100)	2 (100)
Cough	631 (97.5)	132 (99.2)	43 (100)	38 (100)	21 (95.5)	9 (100)	8 (100)	7 (100)	2 (100)	2 (100)	2 (100)
Headache	323 (49.9)	68 (51.1)	20 (46.5)	19 (50.0)	13 (59.1)	4 (44.4)	4 (50.0)	6 (85.7)	1 (50.0)	0 (0.0)	1 (50.0)
Myalgia	294 (45.4)	56 (42.1)	18 (41.9)	16 (42.1)	12 (54.5)	2 (22.2)	4 (50.0)	3 (42.8)	1 (50.0)	0 (0.0)	0 (0.0)
Dyspnea	46 (7.1)	2 (1.5)	1 (2.3)	0 (0.0)	1 (4.5)	0 (0.0)	0 (0.0)	0 (0.0)	0 (0.0)	0 (0.0)	0 (0.0)

PIV = Parainfluenza virus; HCoV = Human coronavirus; RSV = Respiratory syncytial virus; HMPV = Human metapneumovirus; URTI = Upper respiratory tract infection; LRTI = Lower respiratory tract infection; VRI = Viral respiratory infection; Numbers in square brackets ([...]) indicate the total number of positive cases per virus.

## Data Availability

All data is available within the manuscript.
